# Novel CAD–CAM fabrication of a custom-made ball attachment retentive housing: an in-vitro study

**DOI:** 10.1186/s40001-023-01498-5

**Published:** 2023-11-16

**Authors:** Hussein G. El Charkawi, Medhat Sameh Abdelaziz

**Affiliations:** Department of Prosthodontics, Faculty of Oral and Dental Medicine, Future University, Fifth Settlement, End of 90 Street, Cairo, Egypt

**Keywords:** CAD–CAM–PEEK attachment, Measurement of retention, Custom-made attachment

## Abstract

**Purpose:**

This study aims to evaluate the digitally designed ball attachment housing in its initial retentive force and after 2 years of simulated clinical use and to compare it with the regular nylon ball attachment housing.

**Materials and methods:**

Twenty implants with their corresponding ball abutments (diameter 4.5 × 4.0 mm) were inserted in resin blocks. They were divided into two groups. In Group I, ten ball abutments each received their corresponding conventional attachment with nylon rings. In Group II, ten ball abutments received the novel CAD–CAM polyetheretherketone ball attachment housing. A universal testing machine was used to measure the retention force. The achieved maximum values of retention force were recorded at the beginning of the study (initial retention) and after 2 years of artificial ageing (2000 cycles of insertion and removal). Results were statistically analyzed using an independent sample *T* test.

**Results:**

The PEEK attachment housing showed high retention forces (25.12 ± 0.99 N) compared to the conventional attachment with a nylon ring (15.76 ± 0.93 N) in the initial dislodgement test. There was a statistically significant difference in mean retention at the initial retention test and after 2 years of stimulated usage between the two studied groups, *p* = 0.000.

**Conclusions:**

Within the limitations of this study**,** the novel CAD–CAM–PEEK attachment showed high retention characteristics compared to the conventional attachment with nylon rings, initially and after simulated long-term use.

**Supplementary Information:**

The online version contains supplementary material available at 10.1186/s40001-023-01498-5.

## Introduction

Overdenture prostheses have high success rates, as they offer increased retention, stability, esthetic, comfort, bone preservation, and patient acceptance [1]. The attachment is a mechanical device used for the stabilization and retention of the prosthesis. It is composed of two interlocking matrix and patrix parts [2, 3].

A proper attachment selection depends on many factors such as interarch distance, the amount of desired retention force, prosthesis type, inclination and number of implants, and financial options. [4–6]. The attachments are classified as bars, magnets, telescopes, and studs, such as locator, ball and socket, and equator [2, 7–10].

The ball and socket attachment type is widely used in implant overdentures, removable partial dentures, and maxillofacial prostheses. However, there are several inherent deficiencies and shortcomings within this approach. One of which is the loss of retention due to wear of the retaining mechanism of these attachments that require replacement over time [7, 11–13].

The success of implant-retained overdentures depends on many factors to maintain their long-term function. Among these factors is the retentive force of its attachment component. The inevitable movement between the retentive surfaces of an attachment during insertion and removal of the overdenture leads to wear, decreasing retentive forces with time [14–16].

This study adopted a new digital technology workflow for the fabrication of these attachments. Reviewing the literature, none of the previous research has documented CAD–CAM–polyetheretherketone (PEEK) fabrication of the ball attachment housing itself.

The aim of this study was to evaluate the retentive quality of a digitally designed PEEK Ball attachment housing after repeated use. This study compared the retention characteristics of the conventional ball and socket attachment system (housing with nylon ring) with the CAD–CAM–PEEK housing design after 2 years of simulated use. The null hypothesis is that there is no significant difference in retention between the two designs.

## Materials and methods

### Model preparation

A stone model of 4 cm in length, 2 cm in width, and 3 cm in height was constructed in Type III dental stone. This model was duplicated using laboratory addition silicone (REPLISIL 22N, dent-e-con) using a metal flask to build a master mold. This master model was used for subsequent duplication.

Forty identical heat-cured acrylic resin blocks (Acrostone Manufacturing and Import Co.) were constructed. Half of these rectangular heat-cured acrylic resin blocks represent the ridge to which the ball abutment is connected, and the other half simulates the overdenture fitting surface to which the ball attachment housing is connected.

An implant drilling hole was prepared in the resin block with the aid of the dental parallelometer (Ney Surveyor, Dentsply) to ensure that the ball abutment is perpendicular to the horizontal plane. This was done to avoid discrepancies from malalignment of attachment components, which accelerates wear mechanisms [17].

Twenty implants with their corresponding ball abutments were inserted into the standardized resin blocks. A chemically processed acrylic resin (Acrostone Manufacturing and Import Co.) was used to seal the space between the implant and the sidewalls of the drilled hole. Another depression in the simulating overdenture fitting surface blocks was made for the pick-up of the attachment housing.

### Digital ball attachment housing fabrication

**Fig. 1:**
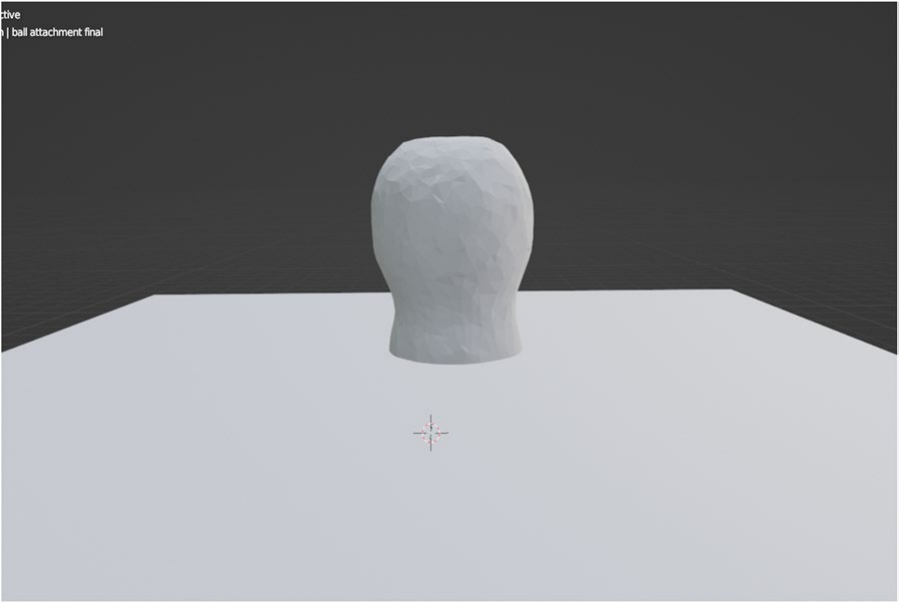
STL file acquired by an optical scanner and representing the ball abutment geometry which is composed of the ball abutment head and ball surface undercut

The digital workflow began with the acquisition of the ball abutment geometry (Ball abutment ISABA 400 diameter 4.5 × 4.0 mm by NeoBiotech Co. Ltd.) by optical scanner (MEDIT i500; MEDIT Corp) (Fig. 1).

**Fig. 2:**
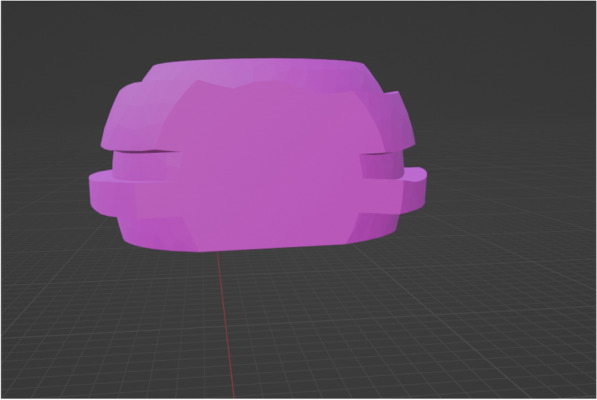
STL file of the outer surface of digitally designed ball attachment housing, the outer surface contains many surface undercuts which will facilitate the future pickup of the housing into the denture

**Fig. 3:**
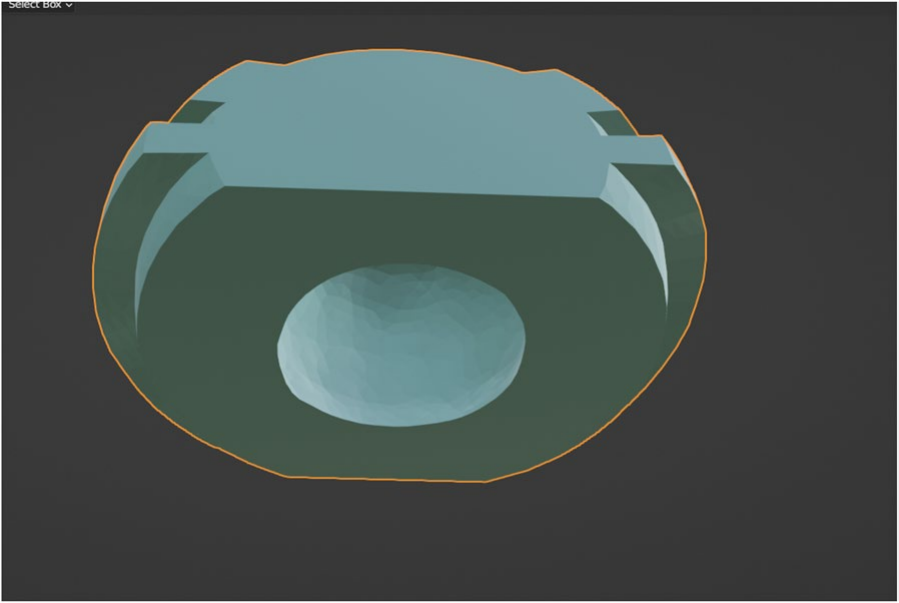
STL file of the inner surface of digitally designed ball attachment housing, the inner surface is a negative replication of the ball abutment housing

The housings were virtually designed using free CAD software (MeshMixer, Autodesk, Inc.). The housing outline was drawn using the select software tool. The housing was given an offset of 0.035 mm to compensate for shrinkage that occurs in the printing of polymethylmethacrylate (PMMA). A 1-mm thickness was given to the attachment housing and then exported in the form of a Standard Tessellation Language (STL) file. A 3D printer (Phrozen sonic mini 4K) was used to print a resin housing which was pressed into a polyetheretherketone (PEEK) housing [5, 18–28] (Figs. 2,3) (Additional file 1: Video S1).

### Groups

Two ball attachment retentive housings were examined in this study; the first is the conventional attachment nylon ring (Group I). The second is the novel digitally designed PEEK attachment retentive housings (Group II).

Ten ball abutments were used for each studied group. This study adopted the methodology utilized by other researchers [5, 10] for the evaluation of the retention force of the selected attachments. The sample size was calculated by G-power software and confirmed by many recent previous studies [5, 10, 29, 30].

**Fig. 4:**
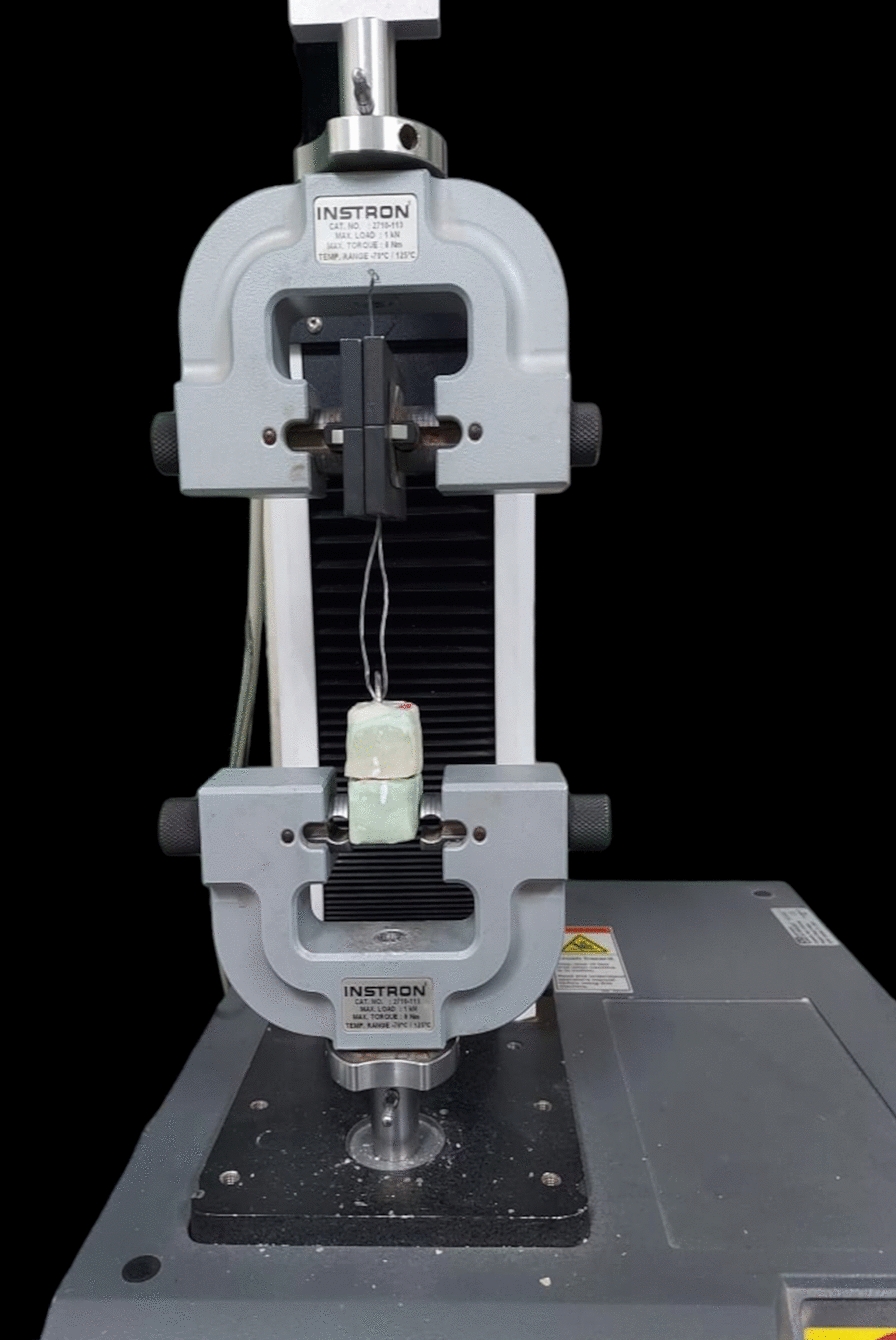
Two resin blocks, one with the ball abutment and the top one with the tested attachments attached to the Instron universal testing machine for measuring retention values

The Instron universal testing machine (Instron, model 3345) was used to measure the retention forces. The abutment base blocks were attached to the lower compartment of the universal testing machine, while overdenture-simulating blocks were attached to the upper compartment of the machine. (Fig. 4) The test was carried out at a crosshead speed of 50 mm/min with a 500 N load cell with removal parallel to the axis of the implant abutment, in the presence of artificial saliva between the abutment and attachments to simulate intraoral conditions.

The retention values were recorded at the initial stage and after 1 and 2 years of simulated clinical use. Each year is simulated with 1000 insertion and removal cycles according to three daily insertion and removal by the patient [5, 10].

#### Statistical methodology

Data were collected and processed on the computer using the SPSS (Statistical Package for Social Science) program for statistical analysis (ver. 25). The parametric statistics were used as the Kolmogorov–Smirnov test of normality showed no significant difference in variable distribution. An independent sample *t* test was used to compare the two studied groups.

## Results

The results of this study showed that the maximum dislodgement force required to pull the novel CAD–CAM–PEEK attachment was 5–6 times that of the conventional nylon ring (25.12 ± 0.99 N) in PEEK housing compared to (15.76 ± 0.93 N) in conventional nylon housing in the initial dislodgement test (Table 1).

**Table 1:** Retention in (Newton) between the studied groups at different times of measurement

	Digital PEEK retention caps (M ± SD) in Newton	Nylon retention caps (M ± SD) in Newton	*P* value
T0	25.12 ± 0.99	15.76 ± 0.93	0.000*
T1	21.84 ± 0.73	13.64 ± 0.70	0.000*
T2	16.76 ± 1.38	12.56 ± 0.69	0.000*

T0: at time of over denture insertion

T1: after 1 year of use

T2 after 2 years of use

^*^Statistically significant (*p* < 0.05)

NS: Statistically not significant (*p* ≥ 0.05)

The retention values were reduced after 1 year to be 21.84 ± 0.73 for PEEK housing and 13.64 ± 0.70 (N) for conventional nylon housing, and then further reduced to be 16.76 ± 1.38 for PEEK housing and 12.56 ± 0.69 (N) for conventional nylon housing after 2 years of simulated use.

Comparisons in retention between the studied groups showed a statistically significant difference in mean retention at the initial retention test (*p* = 0.000*) (Table 1).

**Fig. 5:**
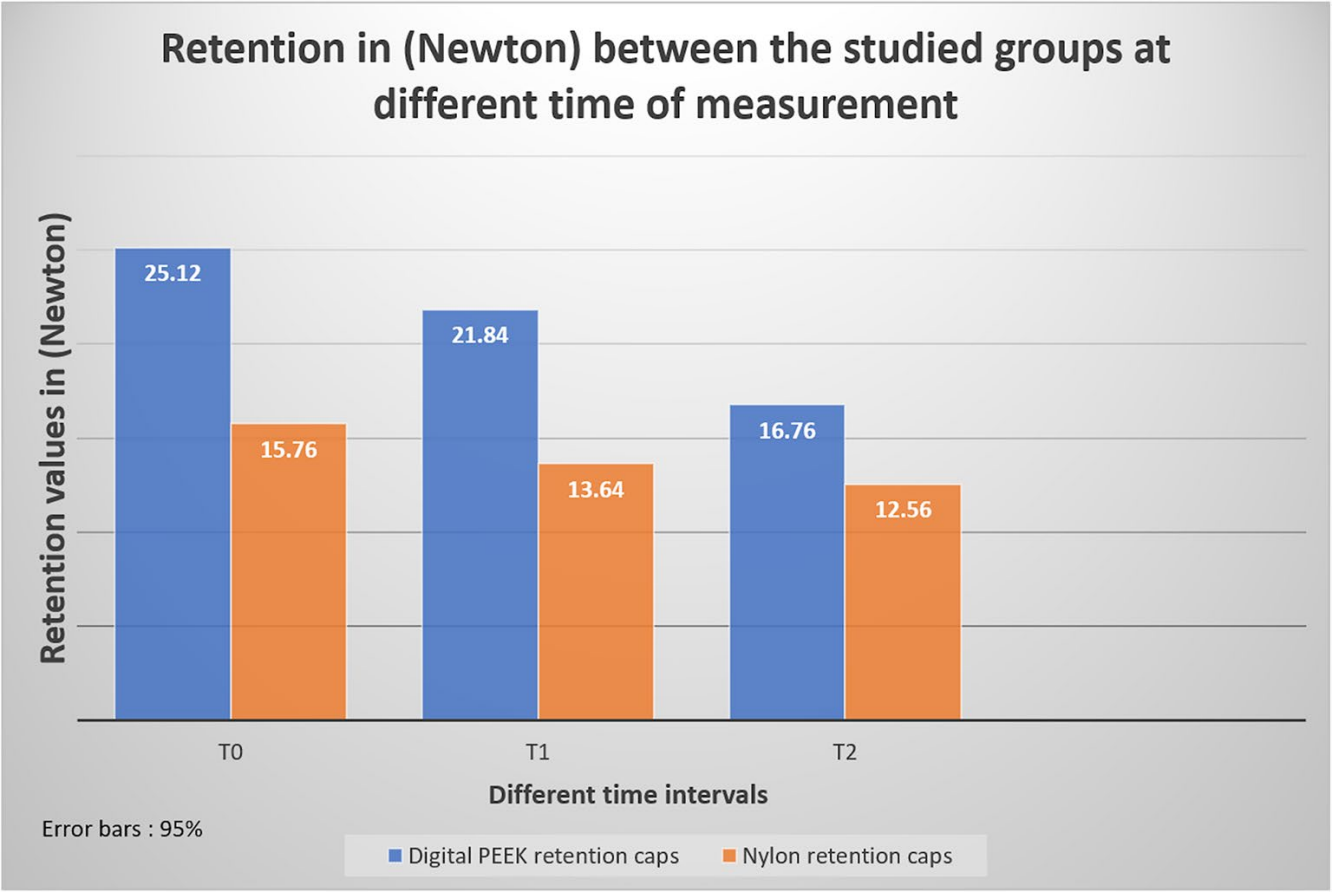
Retention in (Newton) comparing the PEEK and nylon ball attachment housing at different time intervals

CAD–CAM–PEEK attachment housing showed a decrease in retention by 33.28% after 2 years of use, while nylon caps showed a reduction in retention after 2 years of use by 20.30% compared to primary retention (Table 2) (Fig. 5).

**Table 2:** percentage of retention loss in (Newton) between the studied groups at different times of measurement primary retention vs 1, 2 years of use

	Digital PEEK retention caps	Nylon retention caps
	Percentage change	
T1 vs T0 (%)	− 13.05	− 13.45%
T2 vs T0 (%)	− 33.28%	− 20.30%
T2 vs T1 (%)	− 23.26%	− 7.91%

T0: at the time of over denture insertion

T1: after 1 year of use

T2 after 2 years of use

## Discussion

Two challenges in the fabrication of the digital PEEK ball attachment retentive housing were faced. The first was the use of computer-aided design (CAD) to design a virtual attachment retentive housing that conforms to the actual geometry of the existing ball abutment and provides acceptable retention of the prosthesis. The second was how to use the currently available computer-aided manufacturing (CAM) techniques for the attachment fabrication with suitable material [5].

Multiple digital technologies have also emerged in the perspective of the digitization, modeling, designing, and fabrication of different implant-anchored prosthetic components. Despite the great progress and popularity gained by CAD and CAM technology in most dental specialties, such as fixed and removable prosthodontics, aesthetics, and dental implantology, its role in fabricating small and complex geometry components has been, to date, limited [24].

According to the author’s knowledge, none of the previous studies in the literature have dealt with digital fabrication of the ball attachment housing itself. This novel study used digital technology to fabricate a customized ball attachment retentive housing to solve the problem when these attachments are no longer available in stock due to production policies of the manufacturing companies.

The designed attachment housing was 3D printed in resin then pressed into PEEK as 3D printing technologies have many advantages, such as the ability to manufacture complex geometries of small items with no waste of the materials [27]. While this technology has certain limitations, such as the need for a skilled professional with good computing skills [28, 31].

The attachment housing was fabricated from PEEK material as PEEK has many advantages, such as excellent mechanical properties, wear resistance, stability at high temperatures, and biocompatibility [32]. Several in vitro studies and clinical reports suggested that PEEK could be suitable for CAD–CAM fabrication of many fixed and removable dental prosthetics [25]. In a study by Qin et al., it was reported that the use of PEEK material as an attachment reduces the stresses around the abutment teeth and on the edentulous ridge. However, PEEK has an opaque and greyish color, reducing its aesthetic quality [32–35].

The retentive force for each design was measured using a Universal Testing Machine (Instron), which is an evidence based valid method used by many previous studies to measure the retentive forces of different attachments [5, 10, 17, 23].

The results of this study showed that the novel CAD–CAM–PEEK attachment showed higher retention forces than the conventional attachment in the initial testing condition and after simulated use of 2 years. However, both showed a significant reduction in the recorded values after 2 years of use. This could be due to wear and surface topography changes due to continuous friction between the ball abutment and the attachment housing [5, 36, 37].

The significant reduction of retentive values by 33.28% in PEEK attachment after 2 years of use, while nylon caps showed a decrease in retention after 2 years of use by 20.30% compared to primary retention. This could be attributed to the properties of the material from which the attachment housing is fabricated. In elastic materials such as nylon, the wall of the cap is compressed and then returns to its original shape, while in rigid materials such as PEEK, there is outward flex of the wall of the cap. These results are in agreement with previous studies [5, 36–38].

The result of our study agrees with the results of a previous study conducted by Nassar and Abdelaziz [5] who compared the retention force of PEEK and nylon retentive clip of bar attachments and concluded that PEEK clips have comparable or even better retention in comparison to nylon ones due to its high resistance to surface alteration and wear.

On the contrary, a study conducted by Abdelaziz [3] have proved that PEEK locator attachments showed higher retention loss in comparison to Nylon one and this could be attributed to the type of PEEK used in their study and the amount of filler incorporated in this material.

Optimal retention is the level of retention that allows a patient to easily manipulate a prosthesis into position and remove it without dislodgment during normal use [39, 40]. The minimum accepted retention force of different attachment systems for implant-retained overdentures was reported to range from 3 to 8 N [41]. The retention values of PEEK attachment housing recorded in this study were higher than the minimum accepted attachment retentive values. This is in accordance with a study conducted by Abdelaziz et al., who tested the initial retentive values of ball and socket and locator attachments and recorded 15 N and 14 N, respectively [10]. Another study conducted by Nassar and Abdelaziz reported that the initial retentive force of PEEK and Nylon bar attachment clips were 42 N and 16 N, respectively [5]

In this study, lateral forces affecting the retention throughout the chewing process were not simulated in the testing conditions, which is considered as one of the limitations of this study [42]. However, Tehini et al. tested different attachment retentive values after chewing simulation cycles and concluded that chewing simulation did not demonstrate any significant effect or correlation to the attachment retentive values [43].

### Clinical significance

The introduced ball attachment retentive housing could be indicated when a small custom-made attachment is essential to use due to space limitations, unavailability in stock, and in situations when the prosthesis requires high retention qualities, as in a minimum number of implants or in maxillofacial obturator prosthesis.

## Conclusions

Within the limitations of this study, it could be concluded that both attachment housings fabricated from PEEK and Nylon differed in retention values at initial delivery and after simulated use of 2 years. However, the CAD–CAM-fabricated PEEK attachment housing demonstrated the highest retentive values. Both retentive housings exhibited reduced retention after simulated use.

### Supplementary Information

**Additional file 1.** A video demonstrating the digital workflow of the design of the ball attachment retentive insert.

## Data Availability

The data sets used and/or analyzed during the current study are available from the corresponding author on reasonable request.
